# Supplementary material for: The Impact of Intolerance of Uncertainty on Negative Emotions in COVID-19: Mediation by Pandemic-Focused Time and Moderation by Perceived Efficacy
Source: Int J Environ Res Public Health. 2021 Apr 15;18(8):4189. doi: 10.3390/ijerph18084189 (PMC8103505; doi:10.3390/ijerph18084189)
Supplement: Supplementary file 1 [file ijerph-18-04189-s001.zip › ijerph-1179264-supplementary.pdf]

Table S1 Correlations among three negative emotions.

Variables	*M* ± *SD*	1	2	3
1 Fear	2.71 ± 1.68	1		
2 Anxiety	3.38 ± 1.78	0.668***	1	
3 Depression	2.66 ± 1.67	0.697***	0.699***	1

Note: N = 1022. ***, $p < 0.001$.
